# Pterygoid Anchorage of Subperiosteal Implants: An Overview and Case Report

**DOI:** 10.7759/cureus.85175

**Published:** 2025-06-01

**Authors:** Antoine Diss, Augustin Lerebours, Cyrille Grébonval, Laurine Birault

**Affiliations:** 1 Dentistry, Institut du Sous-Périosté, Nice, FRA; 2 Dentistry, Biotech Dental, Nice, FRA; 3 Biomedical Engineering, Glad Medical, Salon-de-Provence, FRA

**Keywords:** additive manufacturing, basal implant, computer-aided design and computer-aided manufacturing (cad-cam), dental implant, full-arch rehabilitation, graftless implantology, immediate loading, pterygoid implant, severe bone atrophy, subperiosteal implant

## Abstract

Severely atrophic maxillae (Cawood and Howell Class V-VI) often prevent conventional implant placement in the absence of extensive bone grafting, which carries risks, especially in elderly patients. Modern subperiosteal implants, digitally designed and manufactured in titanium using additive technologies, offer a graftless solution by anchoring the implant to basal bone structures such as the canine pillars and the zygomatic buttress.

This case report explores the clinical feasibility of adding pterygoid anchorage to increase subperiosteal implant stability. A 71-year-old edentulous female with extreme maxillary atrophy received two custom subperiosteal implants designed with fixation points in the canine, zygomatic, and pterygoid regions. The digital workflow included cone beam computed tomography (CBCT) imaging, digital smile design, and implant modeling with a triply periodic minimal surface (TPMS)-gyroid structure to promote osseointegration. The implants were manufactured using Direct Metal Laser Sintering in Ti-6Al-4V ELI titanium and loaded immediately with a provisional titanium-reinforced polymethyl methacrylate (PMMA) bridge.

Surgical placement included the insertion of bicortical pterygoid screws that penetrated the pterygomaxillary suture, so increasing posterior support and countering the occlusal forces associated with anterior cantilevers. Postoperative results showed excellent implant stability and patient satisfaction.

This report confirms that pterygoid anchorage is a viable addition to subperiosteal implant design. It offers improved mechanical stability and expands treatment options for patients with extreme bone loss, without requiring bone grafting. Further biomechanical and long-term studies are recommended to validate these promising findings.

## Introduction

Patients classified under Cawood and Howell [1] classes V-VI encounter challenges in achieving permanent prosthodontic reconstruction via dental implants due to severe maxillary atrophy, particularly in the premaxillary region [2]. Traditional methods used to address this issue include extensive maxillary sinus lifts with the insertion of bank bone or autologous bone grafts [1]. However, autografts present notable drawbacks, for example, the requirement for a secondary surgical site and the morbidity associated with bone harvesting [3]. Additionally, elderly patients are often less suitable for bone augmentation due to lower metabolic rates and decreased regenerative capacity [4]. In situations where bone augmentation is not feasible, alternative approaches include subperiosteal implants, zygomatic implants, and pterygoid implants.

Modern subperiosteal implants are digitally designed using the patient's DICOM and STL files and produced using additive manufacturing from grade 23 ELI titanium. These technological advances facilitate the anchoring of grade 5 titanium osteosynthesis screws onto basal bone structures that were previously considered inaccessible, such as traditionally, the zygomatic process and the canine pillar adjacent to the pyriform aperture of the nasal cavity [5]. Nonetheless, significant stresses are imposed on these implants during mastication, especially in cases of bruxism [6], and it may be advantageous to further optimize this anchorage.

The purpose of this case report is to demonstrate the clinical feasibility of using the pterygoid region as an additional anchorage point for the treatment of extremely atrophied maxillae using subperiosteal implants.

## Technical report

**Figure 1:**
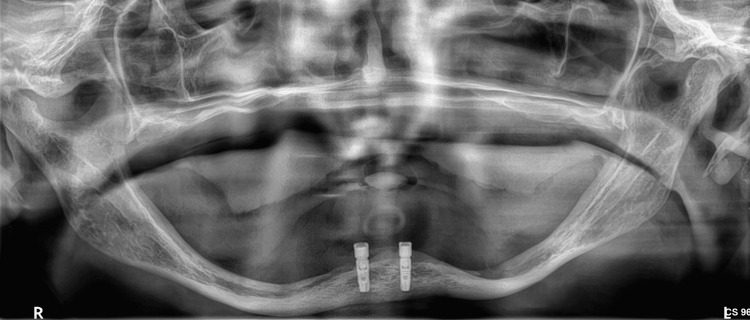
Panoramic X-ray of a 71-year-old female patient. Notice the extreme resorption on the maxilla and the mandible (Class VI in the Cawood and Howell classification)

**Figure 2:**
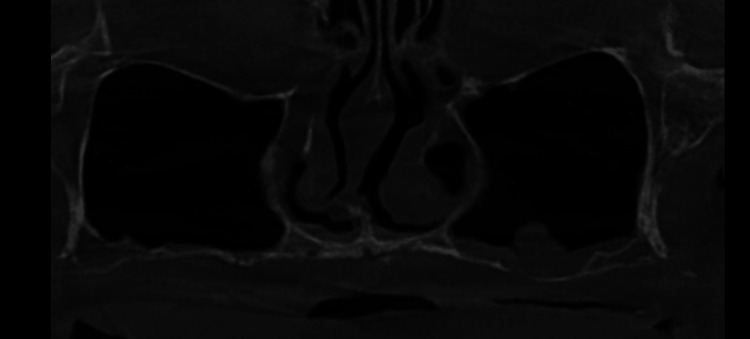
Panoramic reconstruction of CBCT exam. Notice extreme bony atrophy with less than 1 mm bone height under the maxillary sinus and the pterygomaxillary area with basal bone still available

**Figure 3:**
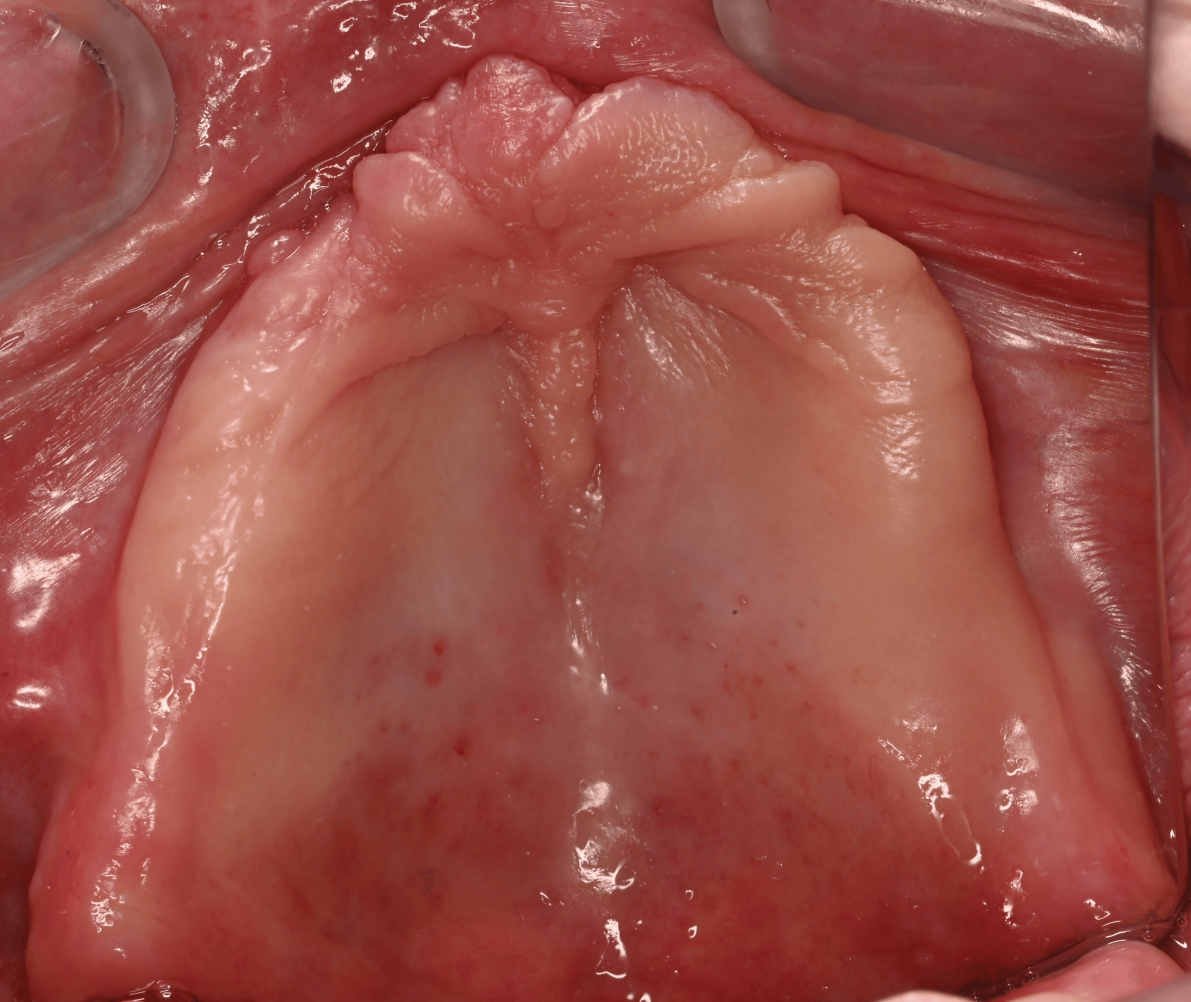
Occlusal view of the fully edentulous maxilla

In March 2024, a 71-year-old female patient presented with a fully edentulous maxilla and expressed a desire for a fixed restoration. The patient had no past or present medical conditions and no ongoing treatment. The patient gave her informed consent after discussing the diagnosis, the outcomes with and without intervention, detailed therapeutic options, and the advantages, inherent risks, and possible complications associated with the treatment. Radiological evaluations, including panoramic X-ray and cone beam computed tomography (CBCT), revealed extreme maxillary bone resorption (Class VI in the Cawood and Howell classification) (Figures 1-3).

Data acquisition

The subperiosteal implant placement process began with a high-quality CBCT examination (CS 9600, Carestream, France) to visualize the patient's zygomatic processes. Next, a dual scan was taken of the complete prosthesis, validated aesthetically and functionally. The last files to be submitted were frontal and profile photos of the patient, including a forced smile, to create a digital smile design. The designer then proceeded to segment the DICOM data, create the digital wax-up, and finally design the implant (HyperMesh 2023®, Altair®, France and Blender 4.3®, Netherlands).

Implant design

The design of these implants required close collaboration between the designer and the dentist during the successive validation steps. First, the designer sited the abutments digitally in relation to the future prosthetic teeth in positions 3, 5, and 7. The goal was for the prosthetic screw channels to emerge in the middle of the teeth of the wax-up.

**Figure 4:**
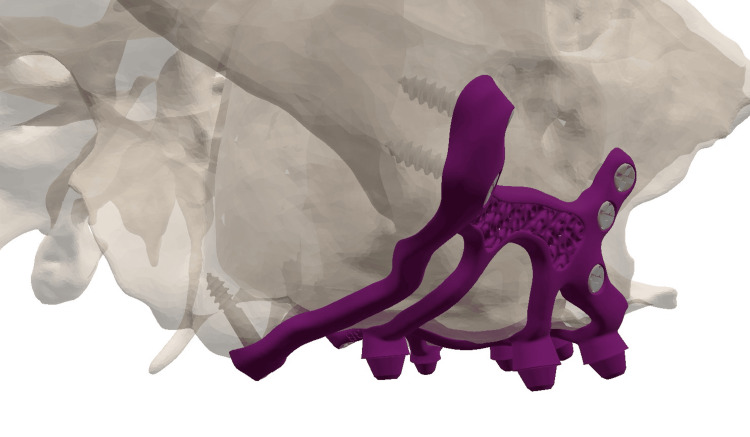
The different anchorages of the subperiosteal implants.

The second step involved placing the fixation screws in favorable anatomical structures: classically three screws (7 mm long and 2 mm in diameter) in the canine pillar (Adapsia®, France), two screws in the zygomatic process (10 mm long, 2 mm in diameter). In this case, two additional screws (2 mm in diameter and 14 mm long) were also placed in the pterygoid region. Screw placement was designed with an upward and inward angle, passing through the maxillary tuberosity and penetrating the pterygomaxillary suture (Figure 4).

Finally, the design concluded with the positioning of the arms, the palatal band connecting the different abutments, and the design of the vestibular implant body joining the paranasal, zygomatic, and pterygoid screws. The implant body received elements to promote osseointegration: a triply periodic minimal surface (TPMS)-gyroid lattice structure with a unit cell size of 2 mm. TPMS lattices have emerged as a distinctive approach to improving the performance of porous scaffold. TPMS structure has a mean curvature of zero in three directions, similar to the natural curvature of trabecular bone, making it an ideal choice for describing the aforementioned biomorphic space [7]. The final design was completed and validated by the dentist before proceeding with bridge production.

Production of the implant

The implant was produced using Direct Metal Laser Sintering technologies in Ti-6Al-4V ELI grade 23. Ti-6Al-4V is also known as grade 5 Titanium while ELI is an abbreviation of “Extra Low Interstitials”. This process promotes implant biocompatibility and osseointegration [8]. Layers of titanium powder were meticulously fused together in DMLS, using a laser beam. This process was repeated layer by layer, allowing the creation of highly detailed and complex implant structures that were previously inaccessible. Two subperiosteal implants (HUG®, Biotech Dental®, France) were printed using this process (TruPrint 1000®, TRUMPF, United Kingdom). The abutments were then machined (Willemin®, Switzerland) to allow for passivity and a perfect fit of the future bridges (which 3D printing does not allow). The arms were polished to avoid peri-implantitis in case of exposure, and the body was sandblasted to improve osseointegration. The micro-roughness of the surface was obtained with 180 µm alumina sandblasted and resulted in a Ra of 1.5 µm. The implant was decontaminated (LLLE6 machine, FISA®, Italy), dried in an oven (Nabertherm®, Germany) and underwent anodization to impart color without impairing osseointegration [9]. The use of color is particularly valuable in case of gingival retraction for esthetic reasons. Finally, the implant was delivered clean but non-sterile and was sterilized in the dental office with a prion cycle in a Class B autoclave to ensure traceability of sterilization.

Production of the provisional bridge

**Figure 5:**
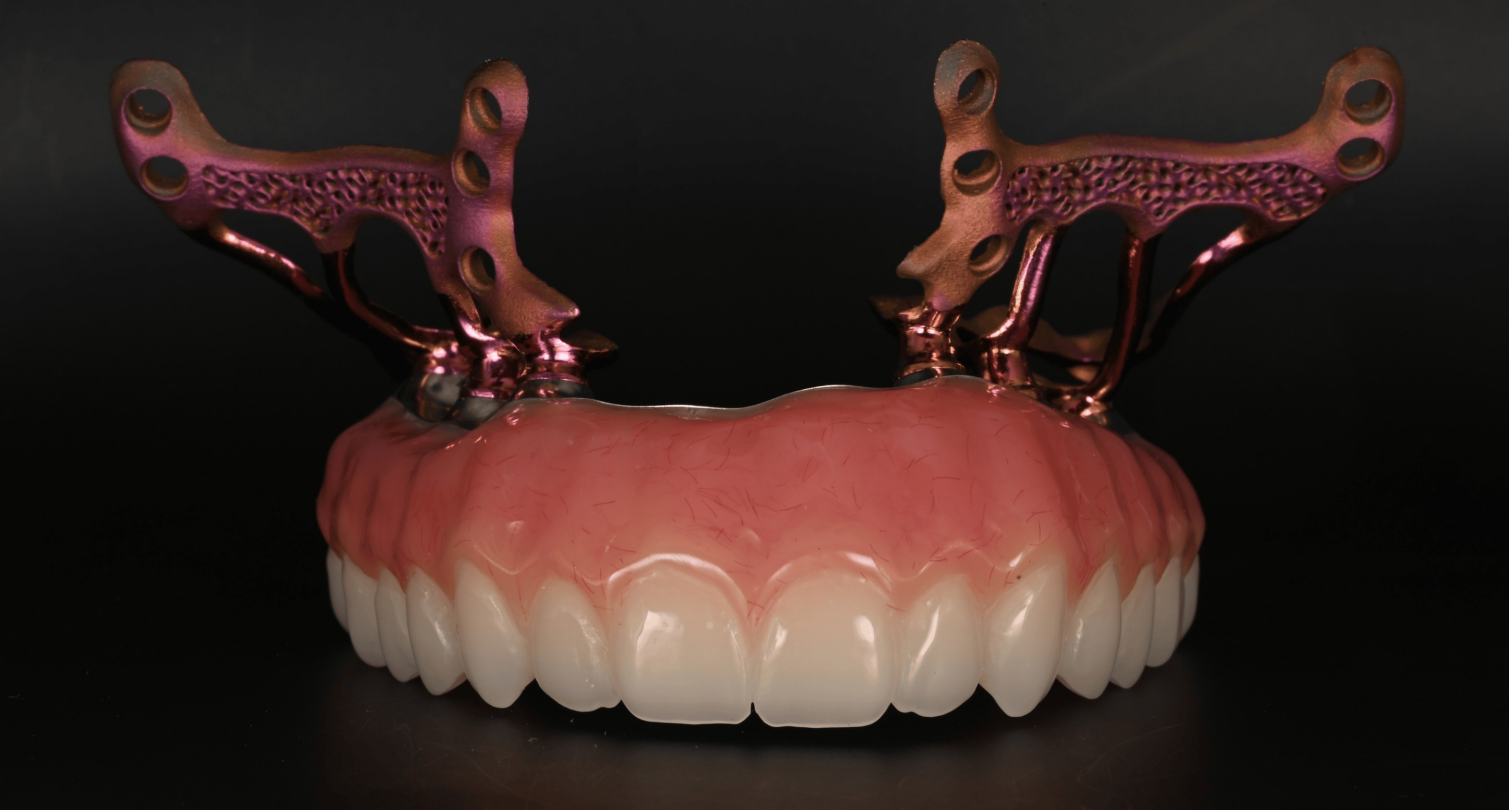
The two subperiosteal implants (HUG®, Biotech Dental) and the fixed provisional bridge with a titanium bar screwed onto the abutments.

**Figure 6:**
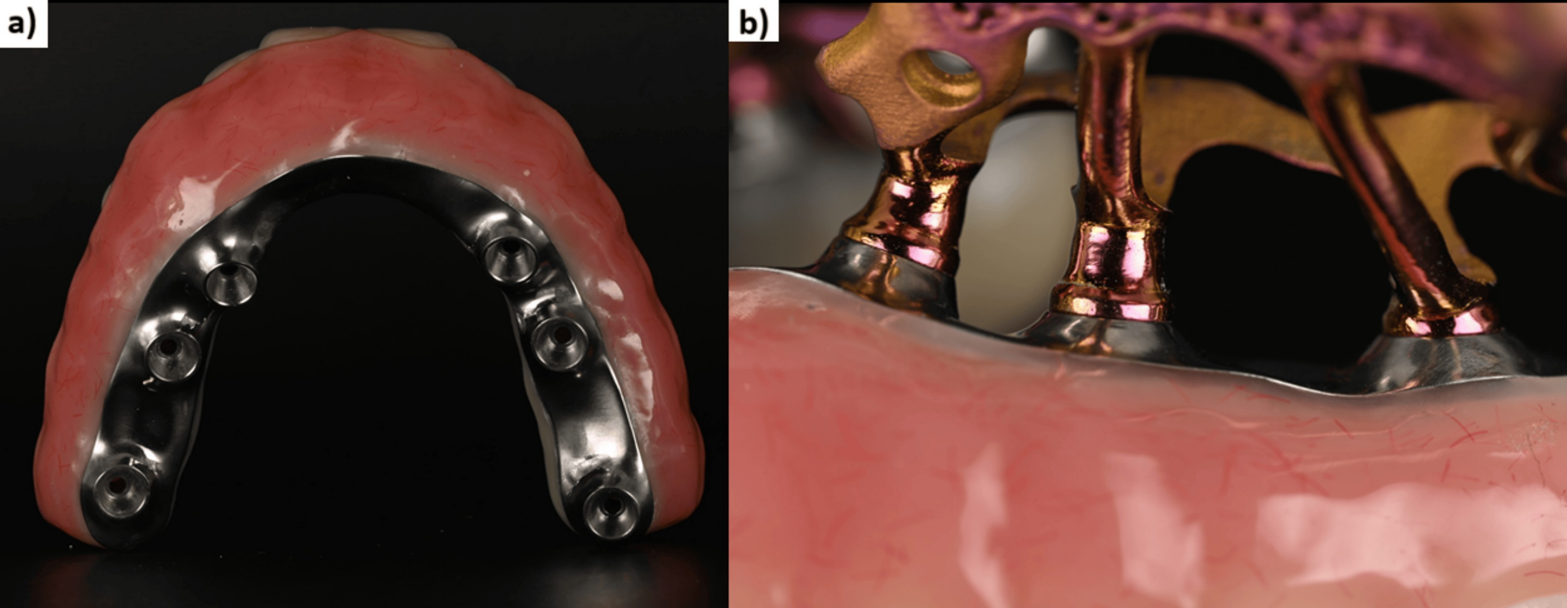
(a) Provisional bridge with machined titanium framework. (b) Focus on the passive fit at the implant–prosthesis connection.

**Figure 7:**
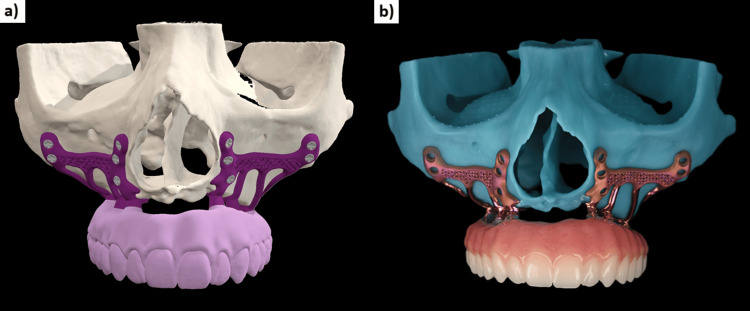
(a) The designed implant and bridge. (b) The finished implant and bridge.

Once the implant design was finalized, the STL files of the implant, its abutments, and the wax-up were used to digitally design the provisional bridge. A temporary fixed bridge machined with a milled titanium framework and embedded in PMMA (Polymethyl methacrylate) was created to ensure maximum solidity of the assembly and to allow immediate loading on the day of surgery (Figure 5). The bridge serves as an external fixator between the two implants. In view of the degree of bone resorption, the prosthesis was constructed with pink false gingivae (Figures 6, 7).

Surgical intervention

Incisions and Flaps Raising

**Figure 8:**
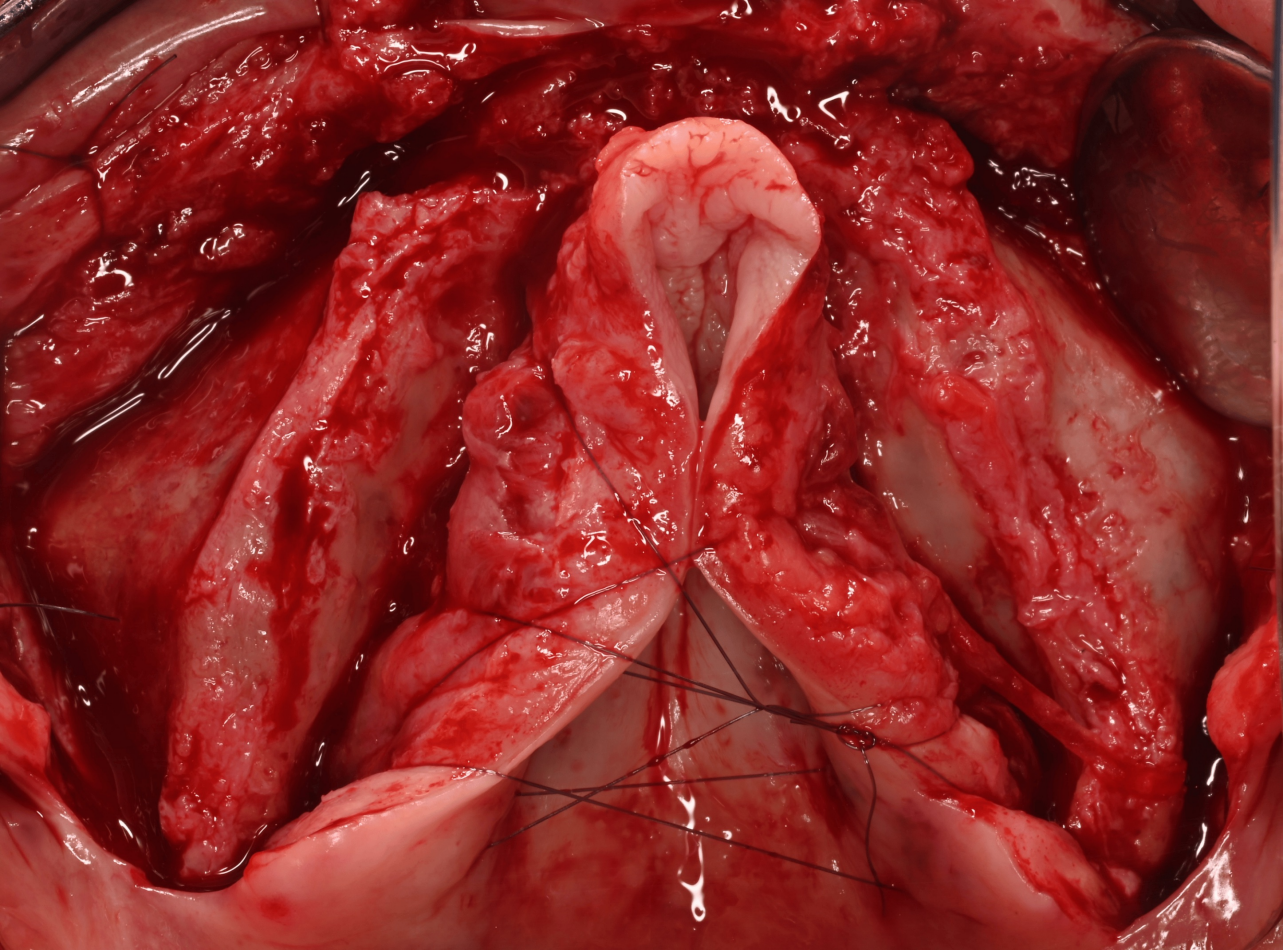
Full-thickness raised flap and exposure of the maxilla: the alveolar crest, the anterior bony wall of the maxillary sinus and the palate

The intervention was performed under local anesthesia in October 2024, in a private dental office in Nice, France. We administered eight cartridges of Articaine with 1:200,000 epinephrine (Septanest®, Septodont®, France). The crestal incision was offset in the palatal direction to preserve a band of attached gingiva on the vestibular side of the abutments and to avoid postoperative dehiscence. The pterygomaxillary ligament was exposed posteriorly. A full-thickness flap was raised to expose the tuberosity, extending broadly along the zygomatic pillar and exposing the infraorbital foramen. The zygomatic process was freed by carefully removing the zygomaticus major muscles, to avoid their fibers being interposed between the implant and the bone. The pyriform openings of the nasal cavities were exposed anteriorly. The palatal flaps were reflected and sutured together to improve site visualization. The pterygoid area was cleared, revealing the greater palatine foramen, preserving the greater palatine neurovascular bundle in the reflected flap, and partially denuding the bony palate (Figure 8).

Subperiosteal Implants Positioning

The implants were then placed and tested for fit. Possible reasons for a poor fit could be design errors (CBCT artifacts, printing issues) or surgical errors (interposition of fibrous tissue, improper use of the cutting guide).

**Figure 9:**
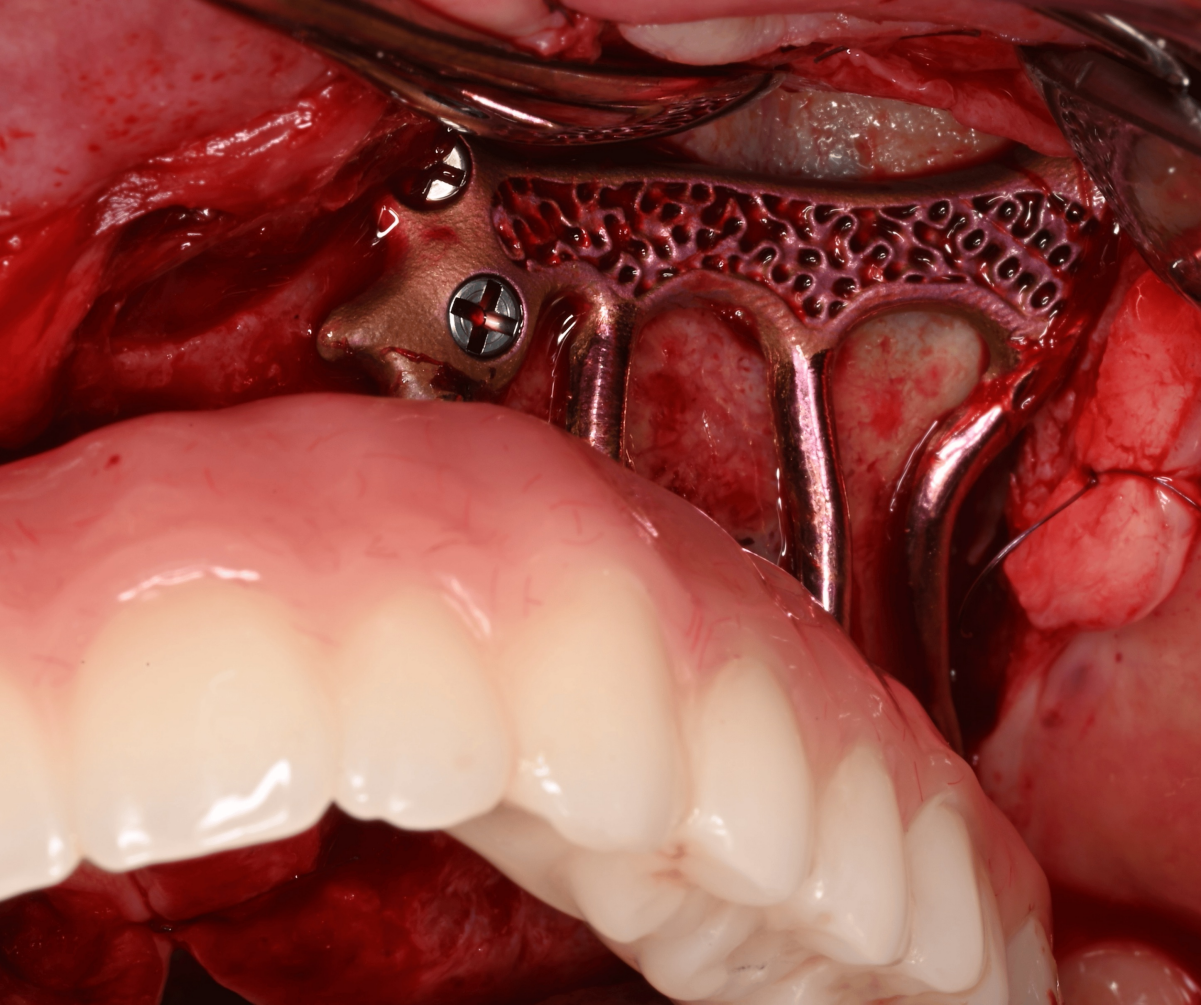
View of the osteosynthesis screws in place at the left canine pillar

Once the two implants were individually well stabilized and properly positioned, the provisional bridge was screwed in place, and the patient was placed in maximum intercuspation. Implant fit was checked again, and the osteosynthesis screws were inserted (Figure 9).

**Figure 10:**
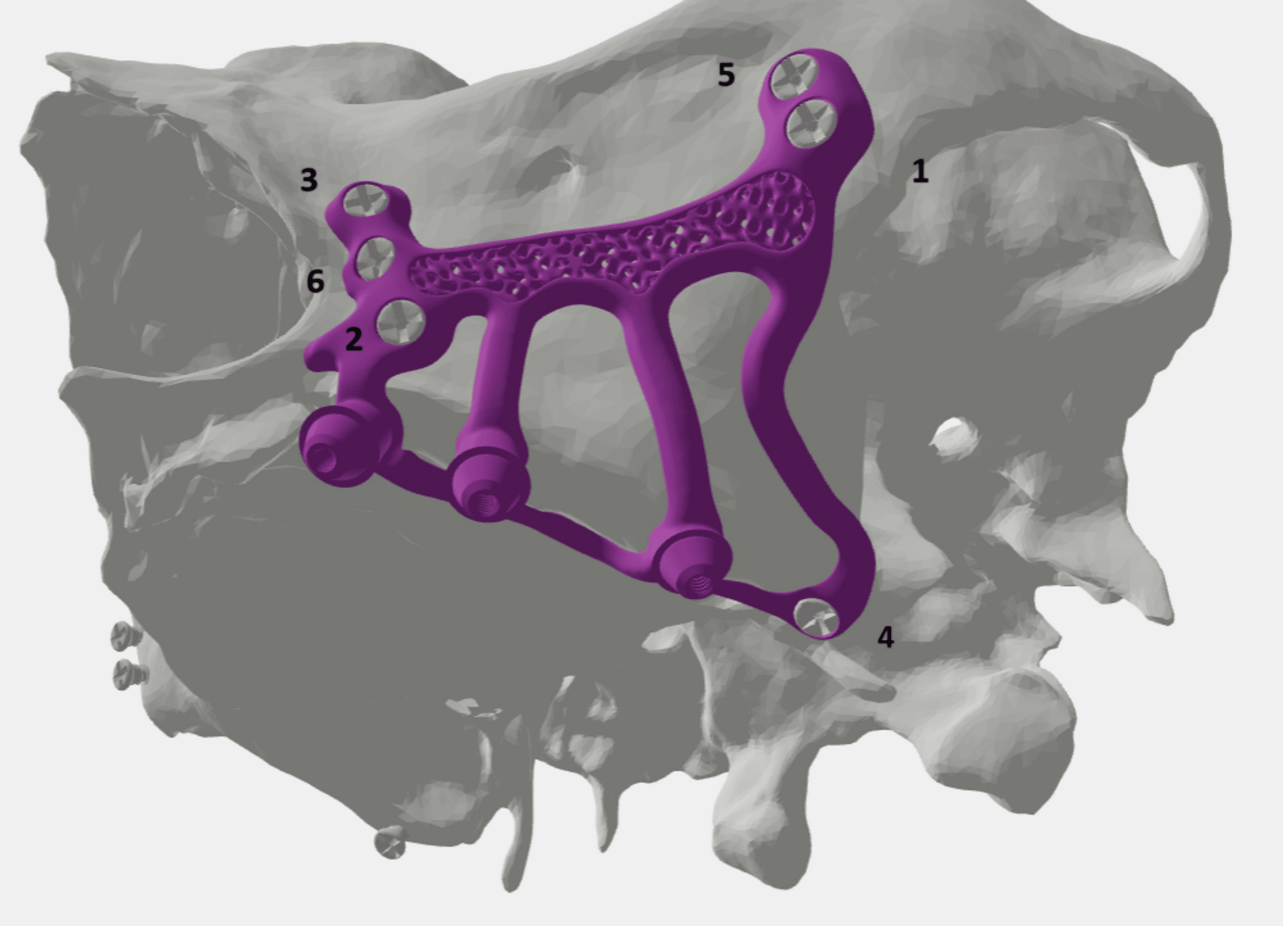
Screw placement protocol; the numbers indicate the screw tightening order for the different anchorage of the subperiosteal implant: the canine pillar, the zygomatic buttress and the pterygomaxillary area

The screws were inserted gradually, until the head made contact with the implant on both sides, then using the final torque (30 N.cm) once all screws were in place. Screws can be inserted in a specific order to ensure a balanced placement of the implants on the bone (Figure 10).

Tissue management

**Figure 11:**
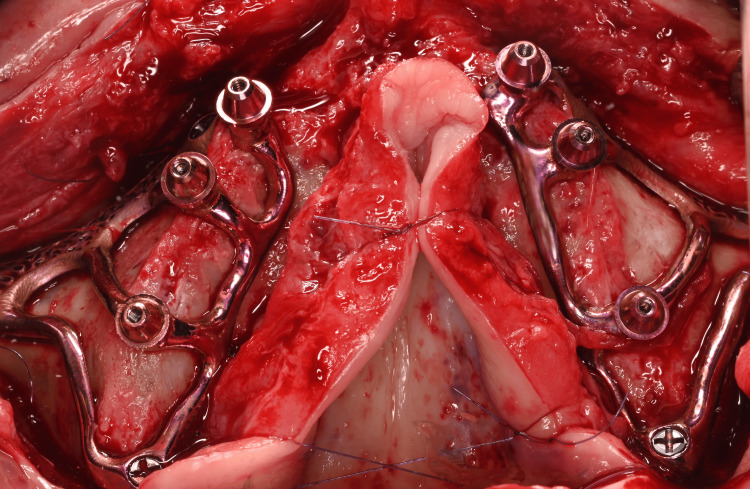
Occlusal view of the two subperiosteal implants (HUG®, Biotech Dental) secured with osteosynthesis screws after removal of the provisional bridge

After implant stabilization, the provisional bridge was removed (Figure 11).

**Figure 12:**
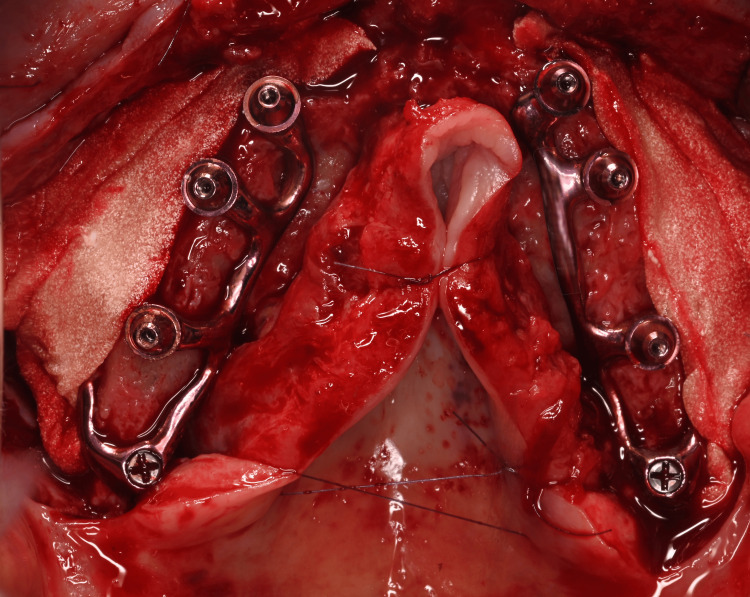
Filling with COLLAPAT II® bone membrane (Biotech Dental), soaked in PRF® (PRF-Process) liquid and covered with membranes.

The outer part of the implant was covered with a bone substitute made of collagen and bovine bone (COLLAPAT II® Biotech Dental), soaked in PRF® (PRF-Process, Nice, France) liquid and covered with PRF® membranes. This process aimed to promote bone growth over the implant (Figure 12) [10].

**Figure 13:**
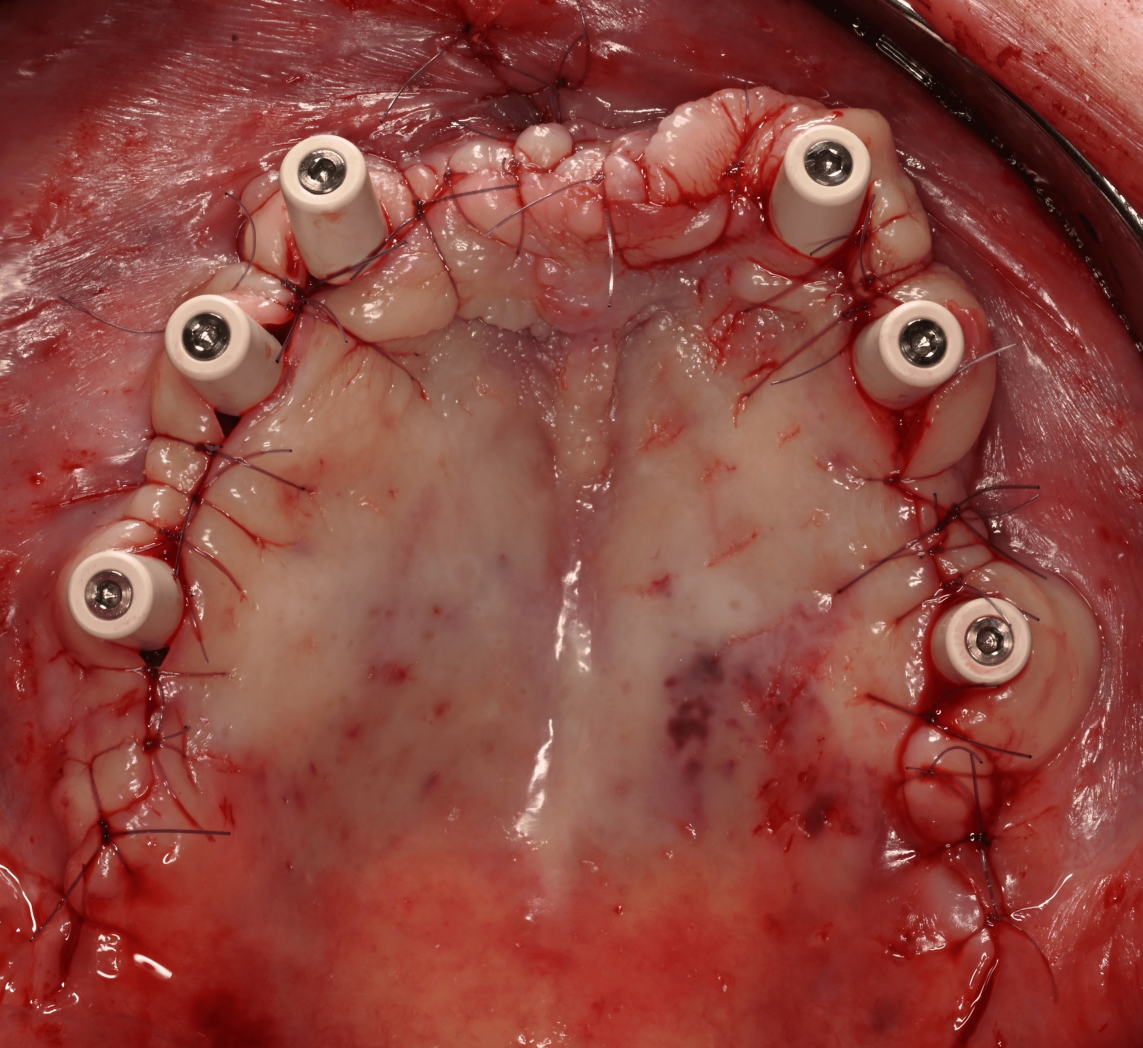
5-0 and 6-0 sutures.

Tall protective caps were screwed onto the abutments, and sutures were inserted using gauge 5-0 or 6-0 resorbable thread (Figure 13).

**Figure 14:**
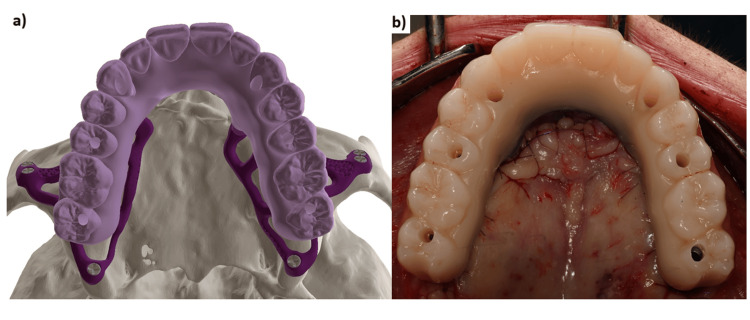
(a) Occlusal view, the digital design and (b) the provisional maxillary bridge screw retained

**Figure 15:**
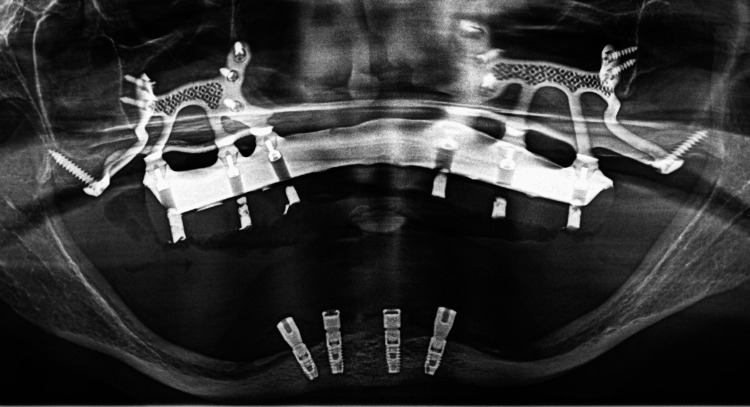
Post-operative panoramic radiograph

The caps were removed, and the provisional bridge was screwed back onto the multi-unit pillars (Figure 14). Occlusal adjustments were made (canine-guided occlusion). The access wells were temporarily sealed, and the patient was able to leave with teeth fixed in place. She was advised not to chew hard food for three months. A panoramic X-ray was taken immediately after surgery (Figure 15).

## Discussion

Modern subperiosteal implants offer advantages, such as a shorter treatment period, reduced cost, and the avoidance of complex, risky surgical procedures [11]. Moreover, there are no reports of resorption of the underlying bone, mobility, or implant fracture, and a 95% survival rate has been documented [11]. However, several studies in the literature involved small patient populations with relatively short follow-up periods [12]. Reported complications include swelling, edema, pain, and implant exposure. Generally, patients have responded positively to the treatment, experiencing enhanced comfort, chewing capability, and prosthetic restoration stability [13]. These results are probably related to the use of new digital technologies, which allow an extremely precise and close apposition of the implant structure to the underlying bone. Patient satisfaction is very good [13]. In a five-year comparative study of zygomatic and subperiosteal implants, Zielinski et al. [14] demonstrated that subperiosteal implants are an alternative to zygomatic implants with identical success rates at five years, and with fewer complications. Subperiosteal implants exhibited superior soft tissue stability with fewer cases of peri-implantitis. Procedural duration was shorter for zygomatic implants; however, subperiosteal implants allowed re-implantation after implant failure, providing flexibility that was unavailable with zygomatic implants.

**Figure 16:**
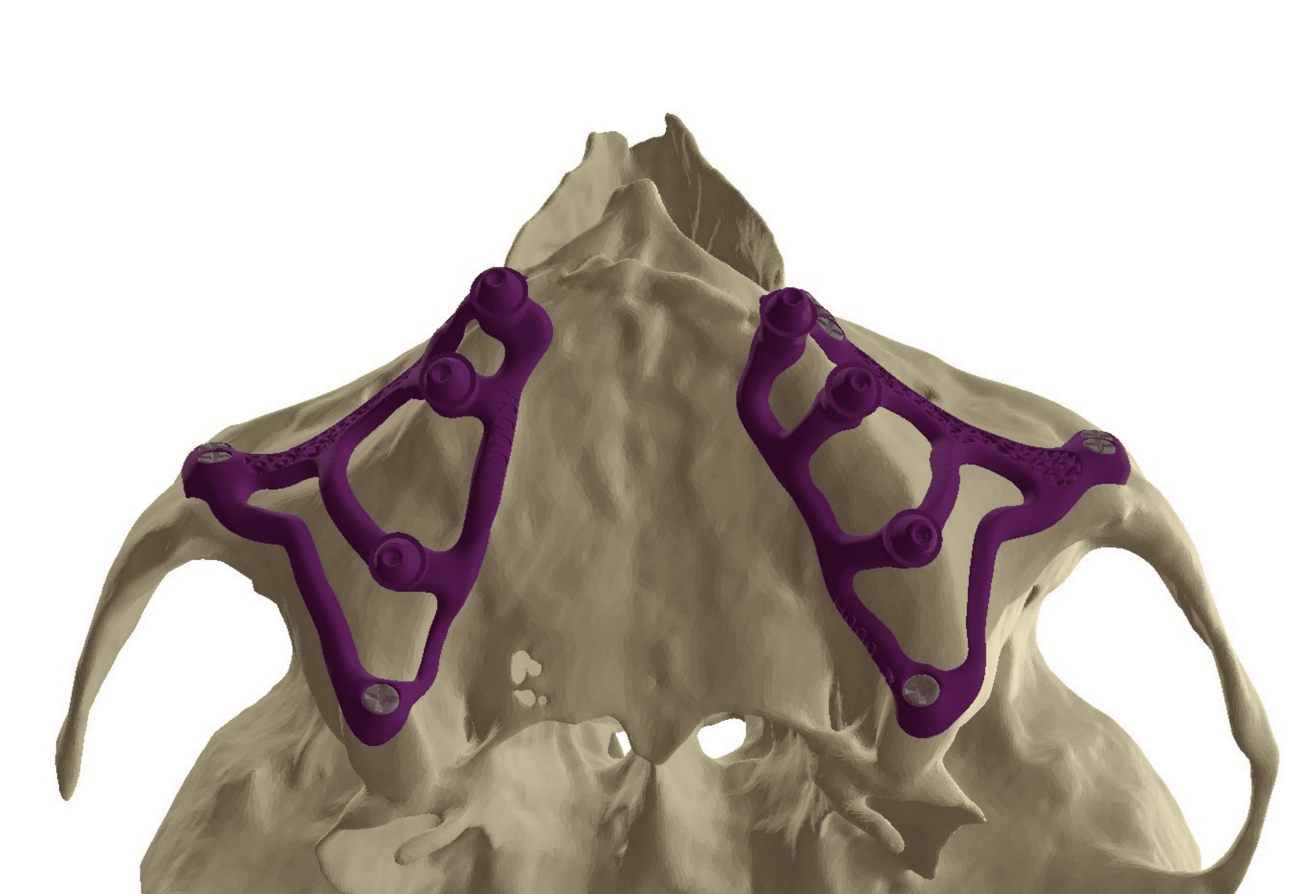
Occlusal view of the two subperiosteal implants

In 1993, Linkow and Ghalili [15] highlighted the necessity of employing anatomical and physiological knowledge to improve the outcomes of subperiosteal implants that extended to the pterygomaxillary suture, located posterior to the tuberosity. The design of the appliance, referred to as a pterygoid subperiosteal implant, features peripheral struts that rest on the lateral or on both the lateral and medial pterygoid plates, the base of the hamulus, the hard palate, the zygomatic buttress, and the canine fossa. The only intrusions on the ridge are for the primary struts [16]. Our aim was to place infrastructural components in areas that are relatively resistant to resorptive phenomena. Many authors of articles dealing with historic subperiosteal implants recommend placing the implant in this area in order to increase bony support [17]. During surgery, we have observed that increased support enhances implant stability prior to screw placement, reducing uncertainty regarding screw positioning. Extending the implant posteriorly onto the palatal area and the tuberosity increased the implant’s support surface and the anterio-posterior spread of 2 mm (Figure 16).

**Figure 17:**
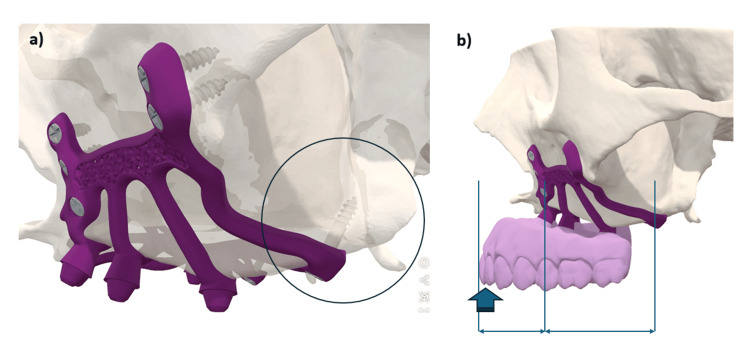
(a) The three anchorage points used for the subperiosteal implant: the canine area, zygomatic areas, and the pterygoid region. (b) The latter anchorage seems to be important as it counteracts the cantilever during incision.

In this clinical case, the implant design extends into this area, but it is additionally anchored bicortically with a 2 mm diameter (up to 2.4 mm), 14 mm long (up to 18 mm), grade 5 titanium screw, that penetrates the pterygomaxillary suture following the same principle as a cylindrical pterygoid implant. Rodriguez et al. [18] analyzed 202 cone-beam computed tomography files of patients with atrophic maxillae and found that the bone density of the pterygoid plate area was three times greater than that of the tuberosity area. A recent systematic review showed a 10-year cumulative success rate of 92.5% for pterygoid implants [19]. From a prosthetic point of view, dental rehabilitation with pterygoid implants has the advantage of eliminating long distal cantilevers, due to the emergence of pterygoid implants in the region of the second molar [20]. Due to bone resorption, we observed a significant anterior cantilever with a considerable overjet of the incisal edges relative to the bone ridge (Figure 17). The pterygoid anchorage helped to counteract these occlusal forces and seems to confer a biomechanical advantage on this implant, which must be demonstrated by finite element analyses. In this article, we have shown that such anchoring is clinically possible.

## Conclusions

Subperiosteal implants are a credible therapeutic alternative for the treatment of patients with completely edentulous maxillae without resorting to bone grafting. These implants are custom-made using computer-aided design/computer-aided manufacturing (CAD-CAM) technologies and are anchored in anatomical areas with no bone resorption: traditionally, the zygomatic process and the canine pillar. It is feasible to supplement this retention with pterygoid anchorage. From a design perspective, extending the design posteriorly increases the supporting surface and facilitates the surgical positioning of the implant. Additional biomechanical studies are needed to validate the value of this anchorage.
